# Melatonin Balance the Autophagy and Apoptosis by Regulating UCP2 in the LPS-Induced Cardiomyopathy

**DOI:** 10.3390/molecules23030675

**Published:** 2018-03-16

**Authors:** Pan Pan, Hongmin Zhang, Longxiang Su, Xiaoting Wang, Dawei Liu

**Affiliations:** Department of Critical Care Medicine, Peking Union Medical College Hospital, Peking Union Medical College, Chinese Academy of Medical Sciences, 1 Shuaifuyuan, Dongcheng District, Beijing 100730, China; 18701545169@163.com (P.P.); ozohom@163.com (H.Z.); slx77@163.com (L.S.)

**Keywords:** UCP2, melatonin, LPS, heart, autophagy, apoptosis

## Abstract

To explore the mechanism of mitochondrial uncoupling protein 2 (UCP2) mediating the protective of melatonin when septic cardiomyopathy. UCP2 knocked out mice and cardiomyocytes were used to study the effect of melatonin in response to LPS. Indicators of myocardial and mitochondria injury including mitochondrial membrane potential, mitochondrial permeability transition pore, calcium loading, ROS, and ATP detection were assessed. In addition cell viability and apoptosis as well as autophagy-associated proteins were evaluated. Melatonin was able to protect heart function from LPS, which weakened in the UCP2-knockout mice. Consistently, genipin, a pharmacologic inhibitor of UCP2, augmented LPS-induced damage of AC16 cells. In contrast, melatonin upregulated UCP2 expression and protected the cells from the changes in morphology, mitochondrial membrane potential loss, mitochondrial Ca^2+^ overload, the opening of mitochondrial permeability transition pore, and subsequent increased ROS generation as well as ATP reduction. Mitophagy proteins (Beclin-1 and LC-3β) were increased while apoptosis-associated proteins (cytochrome C and caspase-3) were decreased when UCP2 was up-regulated. In conclusion, UCP2 may play a protecting role against LPS by regulating the balance between autophagy and apoptosis of cardiomyocytes, and by which mechanisms, it may contribute to homeostasis of cardiac function and cardiomyocytes activity. Melatonin may protect cardiomyocytes through modulating UCP2.

## 1. Introduction

Sepsis, a syndrome of physiologic, pathologic, and biochemical abnormalities induced by infection, is a major public health concern around the world [1]. Sepsis-induced multiple organ dysfunction whose incidence still rising is the major cause of mortality in critically ill patients. The heart and cardiovascular systems are easily and seriously attacked during sepsis [2]. Even many studies have been designed to explore the mechanism and treatment to sepsis-induced cardiomyopathy, its etiology is still unclear and prognosis is poor [3,4]. At present, researchers pay more attention to molecular theory and more and more researchers believe that it is the mitochondria damage causing a series of diseases [5]. Since the heart is the organ that is highly dependent on abundant ATP to maintain its contraction and diastole function, more experiments have proved that mitochondria plays an important role in organ damage during sepsis. Multiple aspects of mitochondria dysfunction, such as disruption of mitochondrial membrane potential, overproduction of reactive oxygen species (ROS), reduction of ATP etc., are thought to influence heart function [6]. 

Mitochondrial uncoupling proteins located in the mitochondrial inner membrane can promote the proton leak across the mitochondrial inner membrane. It is the essential regulator of mitochondrial membrane potential, that disperse the mitochondria proton gradient by translocating H+ across the inner membrane, following with respiratory activity, ROS and ATP generation [7]. Mitochondrial uncoupling protein 2 (UCP2) is the most popular protein in its family, as it can be discovered in various tissues, such as central nerve system, kidney, heart, liver, pancreas, spleen, thymus and macrophages [8]. The wide distribution of UCP2 leads it to have regulation of metabolism, like ROS production, glucose control and immunity and pathologies, like heart failure, diabetes, and cancer [9]. Many studies demonstrated that UCP2 has protective effect on myocardial damage, and down-regulated UCP2 is associated with failing heart [10,11,12,13]. Melatonin, as the best antioxidant and mitochondrial protector, currently, both in vitro and in vivo studies have spoken in favor of high sensitivity of mitochondria to the regulatory effects of melatonin [14,15]. Previous literature in diabetes obesity model indicated that melatonin may regulate UCPs [16]. Nevertheless few studies have been conducted to investigate whether melatonin could influence the uncoupling biological process.

In addition, the further mechanism of uncoupling in heart protecting is unclear. In physiological conditions, autophagy and apoptosis as the programmed cell process play essential roles in cell renewing [17,18]. As the cardiomyocytes have the limited ability to regenerate, continuous cell repairing is critical for maintaining cardiac health, integrity and heart function [19,20]. It includes autophagy and apoptosis to remove and replace the damaged cells and organelles [21]. Even if the mechanisms of autophagy and apoptosis are different, some proteins may be involved in both autophagy and apoptosis progress. In this regard, despite the observation of UCP2 expression in cardiomyocytes, whether it can regulate autophagy and apoptosis still unknown. 

Based on previous studies, we hypothesized that melatonin can influence the UCP2 expression, and thus can protect the cardiac function. To test this hypothesis, both UCP2 knockout animals and in vitro culture of cardiomyoctyes (AC16 cells) were used to study the role of UCP2 in mediating the effect of melatonin in response to LPS insult. 

## 2. Material and Methods

### 2.1. Animal Model and Treatment

Wild C57BL/6J mice were purchased from Beijing Vital River Laboratory Animal Technology Company. All the wild type mice were six-week-old adult male mice, 18–22 g. The UCP2 gene knockout (UCP2-KO) mice were purchased from Nanjing Biomedical Research Institute of Nanjing University. Genotype of knockout mice were detected genomic DNA from the tail by PCR amplification. The gender, week age and body weight of UCP2-KO had no statistical difference compared with their littermates. All the experimental animals had health certificates. All UCP2-KO and their littermates were housed in a constant temperature (20–24 °C) and specific pathogen-free facility. Animals were treated humanely with free access to food and water and maintained under a 12-h light/dark cycle according to guidelines of the Care and Use of Laboratory Animals published by the US National Institutes of Health. All procedures were approved by the Institutional Animal Care and Use Committee (IACUC) of Peking Union Medical College Hospital. 

Mice were divided into the following groups containing 8 mice in each group: (a) wild type (WT) control group, (b) WT + LPS group, (c) WT + melatonin + LPS group, (d) WT + melatonin group, (e) UCP2-KOgroup, (f) UCP2-KO + LPS group, (g) UCP2-KO + melatonin+ LPS group, (h) UCP2-KO + melatonin group. To establish the LPS model, mice were intraperitoneally injected with LPS (Escherichia coli 055:B5; Sigma, St. Louis, MO, USA) at a dose of 20 mg/kg body weight dissolved in 0.2 mL saline. Melatonin was purchased from Medchemexpress (Cat. No. HY-B0075), 30 mg/kg b.w. dissolved in 0.3 mL 0.25% saline. The animals were intraperitoneally injected with melatonin at 3 h, 6 h after LPS administration. Equal amounts of saline were as negative control treatment. According to the results of preliminary experiment, animals were sacrificed twelve hours after LPS injection. Hearts were collected, washed and frozen into the −80 °C refrigerator as quick as possible.

### 2.2. Cardiac Echo Examination

To obtain stable images, mice were anesthetized with 10%chloral hydrate (0.004 mL/g) by intraperitoneal injection and put them on warm pad. They were examined by breathing and toe pinch reflex after anesthesia. Images were acquired by Ultrasonixsonix Tablet (Canada). Echocardiography was performed after LPS and melatonin injection to assess cardiac function. A 20 MHZ transducer was carrying on noninvasive transthoracic echocardiography. Left ventricular ejection fraction and fractional shortening were measured in the short-axis plane, as an index of LV systolic function. End systole and end diastole M-mode in two dimensional were measured in papillary muscle plane. All mice were examined by echocardiography at baseline and 12 h after treatment.

### 2.3. Cell Culture and Treatment 

AC16 cardiomyocytes were purchased from the American Type Cell Culture (ATCC, Manassas, VA, USA). The cells were maintained in DMEM supplemented with 10% fetal bovine serum (FCS), penicillin/streptomycin. Cells were split every 2–3 days. For the experiment, the AC16 cells were treated with 1 µg/mL lipopolysaccharides (LPS) after the pretreatment of melatonin and/or genipin as indicated below. Melatonin and genipin were purchased from Medchemexpress (Cat. No. HY-B0075 and HY-17389). For melatonin treatments, the cells were grown to 80% confluence, and washed by serum-free medium, and then treated with melatonin (100 nM) for 24 h prior to treatment with LPS [22]. For experiments with UCP2 inhibitor, the cells were pretreated with the genipin (50 μm) for 48 h prior to treatment with LPS. The inverted microscope (Olympus IX-61, Tokyo, Japan) was used to observe the morphology of the AC16 cells during the culture process and photographed. Ultra-structural changes were observed by transmission electron microscope (FEI Tecnai Spirit).

### 2.4. Detection mRNA and Protein Expression

Total RNA was extracted from the samples using Trizol reagent (Invitrogen, Carlsbad, CA, USA). Reverse transcription was performed using SuperScript III (Invitrogen). PCR was performed using Eppendorf 5333 MasterCycler Thermocycler (eppendorf, lot: 5333 53658) and Eppendorf Mastercycler ep realplex (eppendorf, lot No.: X226488N). The primers were as followings. UCP2 forward primer, TGCTGAGCTGGTGACCTATG, reverse primer, CCAGGGCAGAGTTCATGTAT; βactin forward primer, GATGAGATTGGCATGGCTTT, reverse primer, GTCACCTTCACCGTTCCAGT. In addition, total protein was extracted from the samples using RIPA Lysis Buffer (Applygen Gene Technology Corp., Beijing, China), and the amount of protein was measured using the Bicinchoninic acid method. Immunoblotting was performed using antibodies against UCP2 (1:1000; sc390189, Santa, U.S.), Beclin1 (1:500; 612113, BD), LC3B (1:500; MBL, PM036), Caspase3 (1:1000; 14220, CST), cytochrome C (1:1000; 4272, CST). The membranes were then washed with TBST three times and incubated with horseradish peroxidase-conjugated secondary antibody (Applygen Gene Technology Corp.). Protein detection was performed using the ECL kit (Applygen Gene Technology Corp.) and images were acquired by exposure to Kodak ×500 film (Midwest Scientific, Valley Park, MO, USA).

### 2.5. Myocardial and Mitochondrial Injury Detection

Myocardial mitochondrial injury detection included mitochondrial membrane potential, mitochondrial permeability transition pore, calcium loading, ROS, and ATP detection. JC-1 mitochondrial membrane potential assay kit (Catlog 10009172, Cayman, Ann Arbor, MI, USA) and Mitochondrial Permeability Transition Pore Assay Kit (Catalog # K239-100, Biovision, Milpitas, CA, USA) were used to detect myocardial mitochondrial injury. Calcium mobilization was detected by FLUOFORTE^®^ Calcium assay kit (ENZ-51017, ENZO, Telluride, CO, USA). ROS was detected by OxiSelect™ Intracellular ROS Assay Kit (STA-342, Cell Biolabs, San Diego, CA, USA). The ATP concentration was quantified by fluorometric detection of ATP using a colorimetric/fluorometric assay kit (Cat. No. MAK190, Sigma-Aldrich, Scotland, UK). All experiments described above were conducted following the manufacturer’s instructions.

### 2.6. ELISA Assay

Nitric oxide (NO) and nitric oxide synthase (NOS) was detected by a double antibody sandwich ELISA purchased from Beyotime (S0024, S0025). Inducible NOS (iNOS), superoxide dismutase (SOD), and troponin (cTnI) was examined by a double antibody sandwich ELISA purchased from USCN (SEA837Mu, SES134Mu, SEA478Mu).ELISA was performed in duplicated wells following the manufacturers’ instructions.

### 2.7. Cell Proliferation and Apoptosis

AC16 cell proliferation and viability at 12h after LPS intervention were determined by MTT Cell Proliferation Assay Kit (10009365, Cayman). Apoptosis of AC16 was measured using the Annexin V-FITC Apoptosis Detection Kit (4830-01-K, RD). AC16 cells were washed twice in PBS and re-suspended in binding buffer (2 × 10^5^ cells/mL). Total 195 μl of the cell suspension was incubated with 5 μl Annexin V-FITC for 10 min at room temperature. After washing with PBS, they were co-stained with propidiumpresidium iodide (PI) and analyzed by flow cytometrycytometer.

### 2.8. Statistical Analysis 

All data were expressed as the mean ± SD. Statistical comparisons among the groups were carried out using a one-way analysis of variance (ANOVA) followed by a LSD or SNK-q protected least significant difference test between any two groups. SPSS 16.0 software was used for all analyses. Values of *p* < 0.05 were considered to be statistically significant.

## 3. Results

### 3.1. Changes in Cardiac Function and Myocardial Damage after LPS Exposure

**Figure 1:**
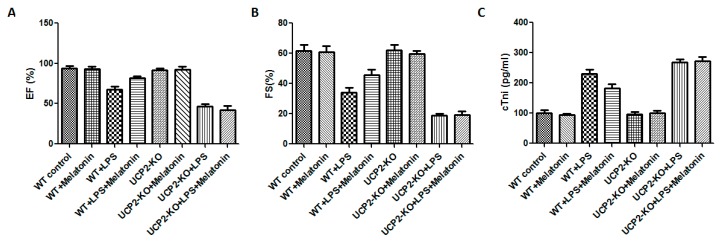
Effect on ejection fraction (EF) and fractional shortening (FS) and tissue injury (troponin, cTnl). EF (Panel (**A**)), FS (Panel (**B**)), and cTnl (Panel (**C**)) were assessed in the animals following injection of LPS and/or melatonin as described in the methods. Vertical axes: Percentage of EF (Panel (**A**)) or FS (Panel (**B**)), or level of cTnl (pg/mL, Panel (**C**)). Horizontal axes: Groups of animals.

Cardiac functions including ejection fraction (EF) and fractional shortening (FS) were measured in all animals. Compared with WT group, cardiac functions of WT + LPS group was significantly affected as demonstrated by significant reduction in EF and FS (Figure 1A,B, *p* < 0.05). In the UCP2-KO animals, LPS (UCP2-KO + LPS group) exposure resulted in further decrease of EF and FS (Figure 1A,B, *p* < 0.05). The mice of WT + LPS + melatonin group had higher EF and FS than that in WT + LPS group (Figure 1A,B, *p* < 0.05). In UCP2-KO mice, however, melatonin could not prevent LPS-induced reduction of EF and FS. Additionally, cTnI was quantified to assess the myocardial damage after LPS exposure. UCP2-KO + LPS and UCP2-KO + LPS + melatonin group had the highest cTnI level than that of other groups (Figure 1C, *p* < 0.05). Furthermore, the cTnI in WT + Melatonin + LPS group was lower than that of UCP2-KO + LPS group, UCP2-KO + LPS + melatonin group and WT + LPS group although there was no statistically significant difference between the groups (Figure 1C, *p* > 0.05).

### 3.2. Alterations in Morphological Characteristics of the Heart Tissue and AC-16 Cell

**Figure 2:**
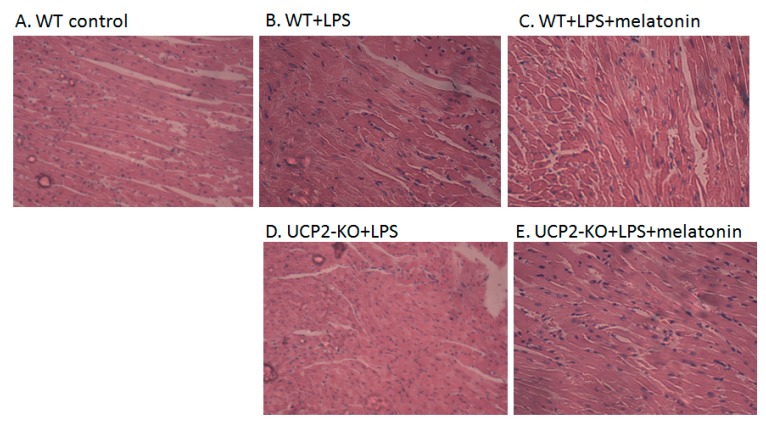

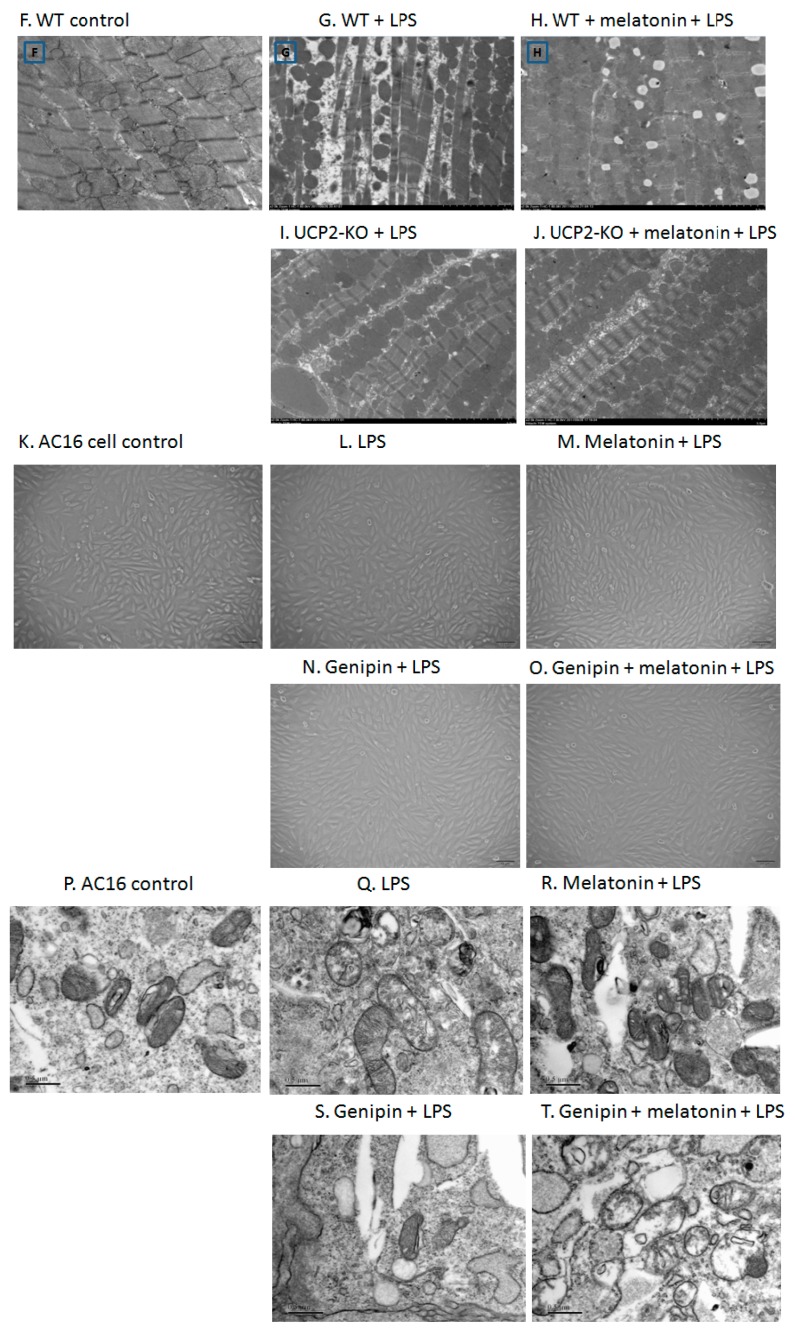
Observation on morphological alteration in heart tissues of animal models or in vitro culture of AC16 cells. Panels (**A**–**E**): H&E staining and histological observation of the heart tissues. Magnification: ×40. Panels (**F**–**J**): Transmission electronic microscopic observation of the heart tissues. Magnification: ×200 Panels (**K**–**O**): Morphological observation of AC16 cells under phase-contrast microscope. Magnification: ×40 Panels (**P**–**T**): Transmission electronic microscopic observation of the AC16 cells. Magnification: ×200.

Figure 2A–J showed the morphological characteristics of the heart tissue collected from the WT and UCP2-KO mouse. The histopathological observation of the heart showed that, compared with the WT group, the heart papillary muscle in the WT+LPS group displayed hemorrhage and edema (Figure 2B vs. Figure 2A). H&E staining of microscopic structures revealed even more severe edema, arrhythmia, and rupture in the myocardial fibers of the UCP2-KO + LPS animals (Figure 2D). Transmission electron microscopic examination of the heart tissue indicated that myocardial fibers in the WT group animals were well ordered and in close proximity, and mitochondria were normal (Figure 2F). In the WT + LPS group (Figure 2G), however, some myocardial fibers were disorganized and loosened, and some even exhibited a scattered distribution. The endoplasmic reticula were dilated and the mitochondria were swelled. Myocardial cells of the WT + melatonin + LPS group (Figure 2H) were improved and autophagosomes were observed in the cells, while it was not observed in the UCP2-KO + LPS group and UCP2-KO + melatonin + LPS group animals (Figure 2I,J).

Figure 2K–O showed morphological alteration of AC16 cells in response to LPS exposure following pre-treatment with melatonin or genipin. After LSP exposure, AC16 cells became sparse, swollen and lost the original spindle shape compared with control (non-treated) cells (Figure 2L vs. Figure 2K). Melatonin pretreatment seemed protect AC16 cells from LPS-induced morphological alteration (Figure 2M), which was abrogated by genipin (Figure 2N,O).Transmission electron microscopic examination of AC16 cells revealed that most mitochondrial membrane was intact, with clear inner ridge and arranged more neatly under the control condition (Figure 2P). After LPS exposure, however, mitochondrial number decreased, the inner crest was in irregular arrangement, morphological change with vacuolar vacuoles, membrane incomplete, and crest rupture or even disappeared (Figure 2Q). Pretreatment of the cells with genipin resulted in further alteration of the aforementioned mitochondrial morphological and structural changes (Figure 2S). In addition, pretreatment of the cells with melatonin could not protect mitochondria from the subcellular structural alterations in the genipin + LPS group (Figure 2T) although melatonin seemed to protect mitochondria subcellular structure changes from LPS insult (Figure 2R vs. Figure 2Q).

### 3.3. UCP2 Expression In Vitro and In Vivo

**Figure 3:**
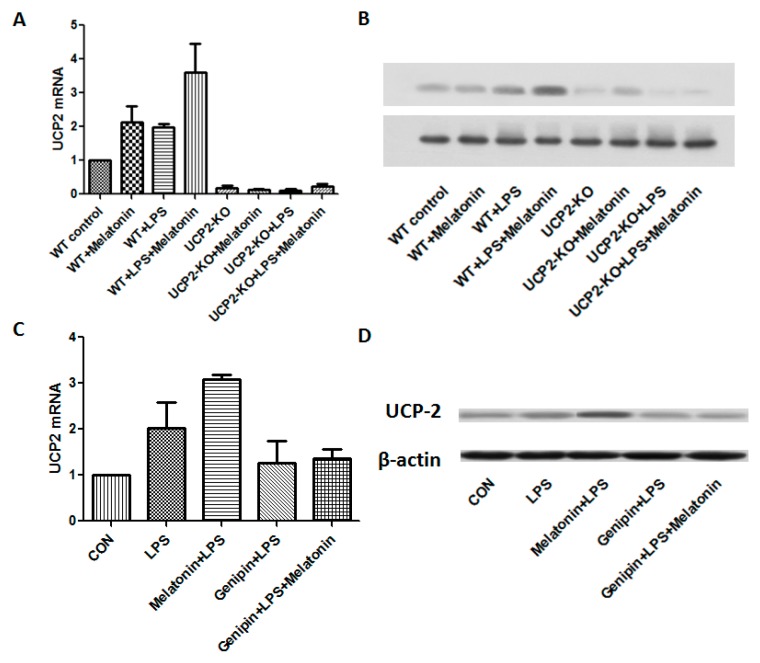
Effect on UCP2 expression in the animal heart tissues and in vitro cell culture. Panel (**A**): Effect on UCP2 mRNA expression in the heart tissues. Vertical axis: Expression of mRNA; horizontal axis: Different treatment groups of the animals. Panel (**B**): Representative image of immunobloting of UCP2 in the heart tissues. Panel (**C**): Effect on UCP2 mRNA expression in the AC16 cells following various treatments. Vertical axis: expression of mRNA; horizontal axis: different treatment. Panel (**D**): Representative image of immunobloting of UCP2 in the cultured AC16 cells under various treatments.

Figure 3A,B showed the changes of UCP2 mRNA and protein expression from different groups of the animals. After LPS injection, UCP2 mRNA and protein were increased. Melatonin further augmented expression of UCP2 mRNA and protein in response to LPS (WT + Melatonin + LPS group, *p* < 0.05). As expected, UCP2 were not expressed in the UCP2-KO mice regardless of treatment. As shown in the Figure 3C,D, both UCP2 mRNA and protein were increased in the AC cells after LPS exposure. Melatonin further significantly augmented LPS-induced UCP2 mRNA and protein expression (*p* < 0.05). In contrast, pretreatment with genipin resulted in slight but not significant blockade of LPS-induced up-regulation of UCP2 mRNA and protein expression (*p* > 0.05).

### 3.4. Mitochondrial Injury of the Myocardial Cells 

**Figure 4:**
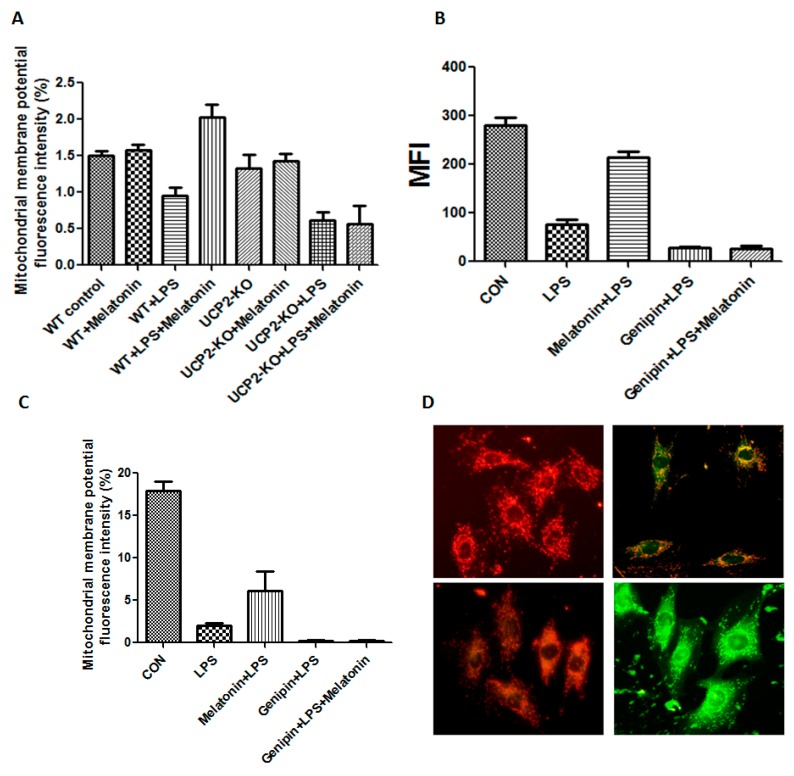
Effect on mitochondrial membrane potential and permeability transition pore. Panel (**A**): Effect on mitochondrial membrane potential in the heart tissues of the animals. Vertical axis: Mitochondrial membrane potential as expressed by fluorescence intensity (%); horizontal axis: groups of animals. Panel (**B**): Effect on mitochondrial permeability transition pore in the cultured AC16 cells. Vertical axis: Mitochondrial permeability transition pore (MFI); horizontal axis: Treatment of the cells. Panel (**C**): Effect on mitochondrial membrane potential in cultured AC16 cells. Vertical axis: mitochondrial membrane potential as expressed by fluorescence intensity (%); horizontal axis: Groups of animals. Panel (**D**): The fluorescence changes in mitochondrial membrane potential. Left upper showed control, left lower showed LPS + melatonin, right upper showed LPS group, right lower showed LPS + genipin group.

The mitochondrial membrane potential of the heart tissue was shown in Figure 4A. Mitochondrial membrane potential was significantly decreased in the wild type animals injected with LPS compared to that in control animals (*p* < 0.05), while it was increased in the animals treated with LPS plus melatonin (WT + Melatonin + LPS group) although it was not affected by melatonin alone (WT + melatonin group). Moreover, mitochondrial membrane potential was further decreased in the UCP2-KO animals injected with LPS (UCP2-KO + LPS group, *p* < 0.05) and melatonin could not prevent the alteration (UCP2-KO + melatonin + LPS group). 

Mitochondrial membrane potential and mitochondrial permeability transition pore were also assessed in the AC-16 cell. As shown in Figure 4B–D, mitochondrial membrane potential fluorescence intensity was significantly decreased in the cells treated with LPS compared with that in control cells (*p* < 0.05), melatonin could block mitochondrial permeability transition pore (MFI, *p* < 0.05, Figure 4B) but not mitochondrial membrane potential (Figure 4C). Pretreatment with genipin resulted in further reduction in both mitochondrial membrane potential and mitochondrial permeability transition pore, and melatonin pretreatment could not prevent it (Figure 4B,C). Figure 4D showed the fluorescence changes in mitochondrial membrane potential. The control group showed red fluorescence due to the high mitochondrial membrane potential. Conversely, mitochondrial membrane potential decreased to show green fluorescence after addition of LPS or genipin. After melatonin intervention, the membrane potential can be obviously increased.

### 3.5. Oxidative Injury in the Heart Tissue

**Figure 5:**
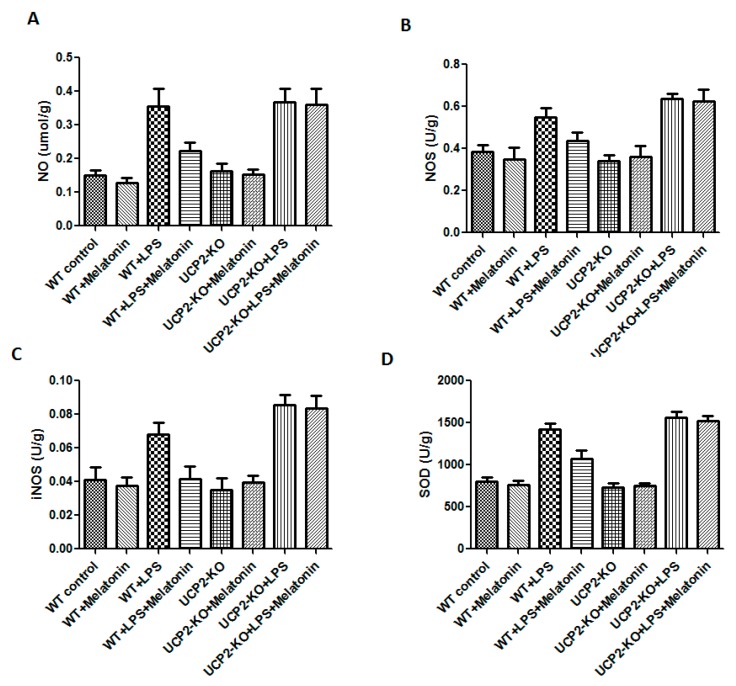
Effect on nitric oxide (NO), nitric oxide synthase (NOS), inducible nitric oxide synthase (iNOS), and superoxide dismutase (SOD) production in the heart tissues. Panel (**A**): NO. Panel (**B**): NOS. Panel (**C**): iNOS. Panel (**D**): SOD.

NO, NOS, iNOS, and SOD were measured in order to assess oxidative injury of the heart tissues. As shown in Figure 5, the levels of NO, NOS, iNOS, and SOD were significantly increased in the animals of WT + LPS group compared with the WT control group (*p* < 0.05). In response to LPS injection, NO, NOS, iNOS, and SOD slightly increased more in the UCP2-KO animals compared to the wild type animals, but none of them was statistically significant.

### 3.6. Effect on the Calcium Loading and Reactive Oxygen Species Production in AC-16 Cell

**Figure 6:**
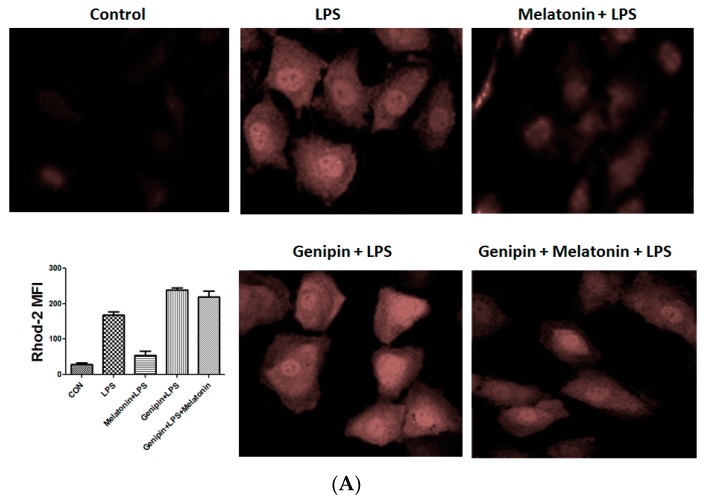

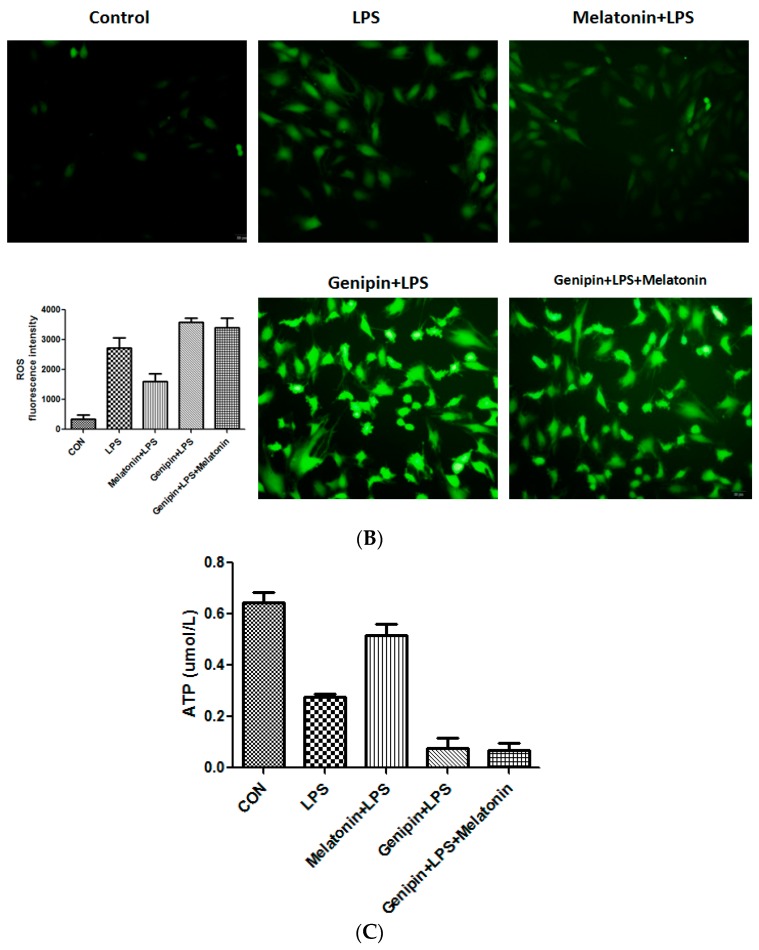
Effect on calcium loading, reactive oxygen species (ROS) and ATP synthesis in the cultured AC16 cells. Panel (**A**): Effect on calcium loading in the cells treated with indicated reagents. Insert: an average of fluorescence intensity. Panel (**B**): Effect on ROS production in the cells treated with indicated reagents. Insert: An average of fluorescence intensity. Panel (**C**): Effect on ATP synthesis in the cells treated with different reagents. Vertical axis: ATP level (µmol/L); horizontal axis: treatment.

Calcium loading and reactive oxygen species (ROS) were examined the AC16 cells following the treatment. As shown in Figure 6, LPS significantly stimulated calcium loading and ROS production, which was significantly blocked by the pretreatment with melatonin (Figure 6A,B, *p* < 0.05). Genipin further augmented LPS-induced calcium loading and ROS production, and melatonin could not block it (Figure 6A,B). Additionally, ATP level was significantly reduced in response to LPS, and melatonin could significantly block the LPS-induced ATP reduction (Figure 6C, *p* < 0.05). In the presence of genipin, however, melatonin could not block the LPS-induced ATP reduction (Figure 6C).

### 3.7. Effect on Cardiomyocyte Viability and Apoptosis 

**Figure 7:**
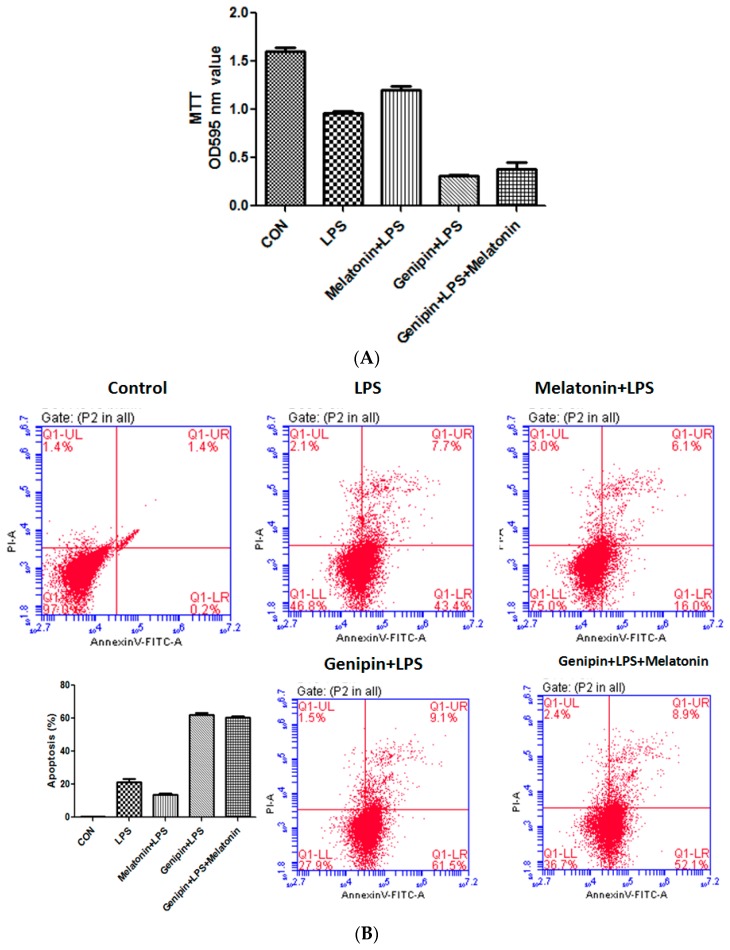
Effect on cell viability and apoptosis in the cultured AC16 cells. Panel (**A**): Effect on cell viability assessed by MTT assay. Vertical axis: OD value; horizontal axis: treatment. Panel (**B**): Effect on apoptosis assessed by annexin-V assay.

Cell viability was examined by MTT assay while apoptosis was assessed by annexin-V staining. As shown in Fig7, LPS significantly reduced cell viability (Figure 7A, *p* < 0.05) but significantly increased apoptosis (Figure 7B,C, *p* < 0.05), and melatonin could partially but significantly block it (*p* < 0.05). Pretreatment with genipin followed by LPS exposure resulted in further reduction in cell viability (Figure 7A, *p* < 0.05) and increase in apoptosis (Figure 7B,C, *p* < 0.05), and melatonin could not block it.

### 3.8. Effect on the Proteins Associated with Apoptosis and Autophagy 

**Figure 8:**
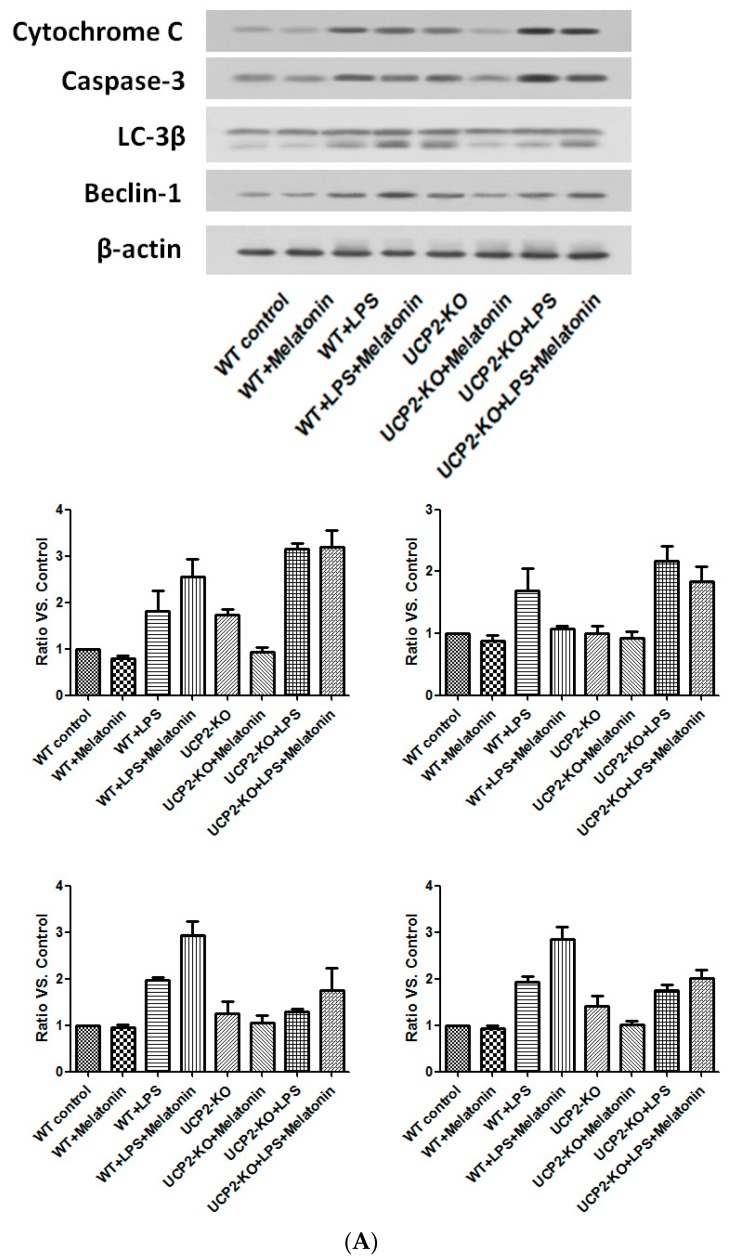

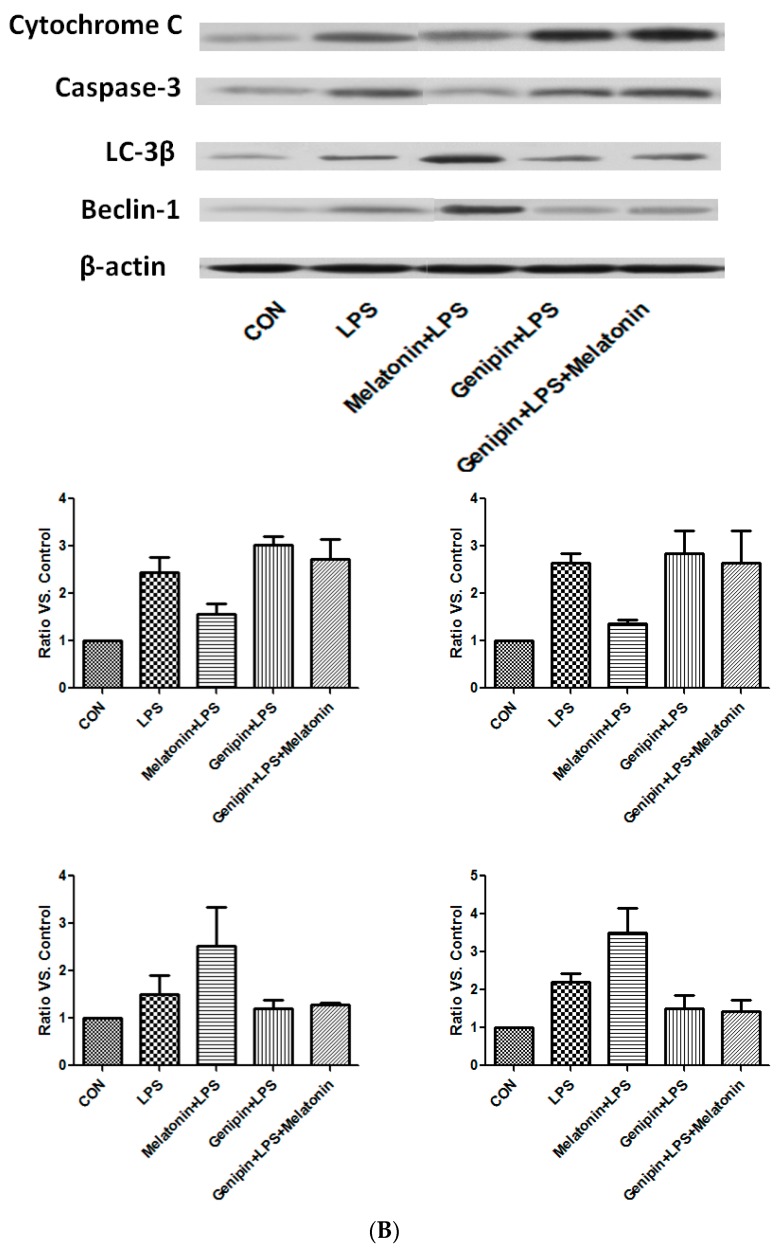
Effect on the expression of apoptosis-associated proteins and autophagy-associated proteins. Panel (**A**): Effect on proteins in the heart tissues of different groups of animals. Panel (**B**): Effect on the proteins in the cultured AC16 cells treated with indicated reagents.

Cytochrome C and caspase-3, the proteins associated with apoptosis, as well as Beclin-1 and LC-3β, the proteins associated with autophagy, were examined by immunoblotting. As shown in Figure 8, cytochrome C and caspase-3 were increased in the wild type and UPC2-KO animals injected with LPS, which was slightly blocked by melatonin (Figure 8A). Similarly, LC-3β and beclin-1 were also increased in both wild type and UCP2-KO mice in response to LPS, which was not affected by melatonin (Figure 8A). 

In the culture of AC-16 cells, LPS exposure resulted in significant up-regulation of cytochrome C and caspase-3 as well as beclin-1 and LC-3β (Figure 8B). Melatonin pretreatment resulted in partial blockade on LPS-stimulation of cytochrome C and caspase-3, but further stimulation on beclin-1 and LC-3β (Figure 8B). Furthermore, genipin could potentiate LPS-stimulation on cytochrome C and caspase-3 expression, but significantly inhibited beclin-1 and LC-3β expression (Figure 8B). Melatonin had no significant effect on those proteins when the cells were pretreated with genipin (Figure 8B). 

## 4. Discussion

The principle findings in our study were as follows. First, LPS up-regulated the UCP2 expression and melatonin further augmented UCP2 expression. UCP2-KO mice had worse heart function after LPS injection. Melatonin could ameliorate heart dysfunction induced by LPS. Second, LPS exposure damaged the AC16 cells (human cardiomyocytes) and melatonin could protect the AC16 cells from LPS-induced cell damage. Cells dealing with a specific inhibitor genipin had been severely attacked in the LPS model, indicating UCP2 can be the protector during sepsis. Then, mitochondrial membrane potential (△Ψm) loss, mitochondrial Ca^2+^ overload, the opening of mitochondrial permeability transition pore (mPTP), and subsequent increased iNOS, NO ad SOD generation as well as decreased ATP production were prevented by UCP2 overexpression. Last but not the least, both the animal and cell models demonstrated that mitophagy protein like Beclin-1 and LC-3β were obviously increased as the UCP2 overexpression, while apoptosis protein cytochrome C and caspase-3 were decreased. Our findings suggested that UCP2 is crucial for protecting against sepsis-induced cardiomyopathy by promoting cardiomyocytes survival through autophagy induction. In addition, melatonin maybe a potential therapeutic drug targeting UCP2 to balance the autophagy and apoptosis.

Sepsis is not only a critical illness in the medical field around the world, but also an unsolved disaster to human. It has been constantly that researchers had created numbers of clinical and experimental studies to seek the treatment protocols [23,24]. LPS is one of the most useful ways to induce septic models in vivo and in vitro. In our study, the structural disorder of the heart tissue after LPS and AC16 cells with LPS were cracking and swelling through light microscope observing, demonstrating that our septic models had been created successfully. These results are also in line with other experimental results [25,26]. Increasing evidences have proved increasing of oxidative damage markers like iNOS, NO and SOD after LPS administration [27], as our study also confirmed this phenomenon. Melatonin acting as an antioxidant can alleviate oxidative stress injury and improve the mitochondria function [28,29,30]. In our study, melatonin groups showed decreased ROS and improved cardiac and mitochondria function apparently compared with LPS groups. Mitochondrial uncoupling proteins located in the mitochondrial inner membrane can promote the proton leak across the mitochondrial inner membrane [31]. UCP2 as the most popular isoform in UCP family, can be discovered in various tissues [32]. Its wide distributions results in the varieties of functions and maybe organ protection in pathological condition. Precious studies had illustrated that UCP2 can be up-regulated in sepsis [33,34]. Our experiment also suggested the levels of UCP2 mRNA and protein were raised in septic status. However, Michael, et al. found UCP2 mRNA increased but no UCP2 protein detection in their study [35], they had no definite explanation of the discordance, but they also proved UCP2 had no negative influence on cardiac mechanical efficiency. To further explore and confirm whether UCP2 had effect on sepsis-induced cardiomyopathy, we use UCP2 knocked out animal model in vivo and genipin to be the UCP2 specific inhibitor in vitro like other research [36,37]. Our results demonstrated that UCP2 blocking can bring catastrophic injuries to myocardial cells in sepsis, which was similar to precious study [25]. Both animals’ cardiac cells and AC16 cells were obviously swelling and crushing. At the molecular level, the decreased MMP and dissipation of membrane potential made ROS increased abnormally and ATP reduced. From the functional level of the heart, ultrasound results displayed the failure of cardiac pump under septic conditions. As it was clear that UCP2 played a protective role in cardiovascular disease in our research, upregulating the UCP2 expression could be a potential therapeutic target. Melatonin acting as one of the most effective antioxidants is mitochondria-targeted, it can protect the cells from oxidative or nitrosative damage [38,39]. In diabetic obese animals’ model, researchers found melatonin can reduce hepatic mitochondrial dysfunction and augment ATP production [40]. However in their results, melatonin reduced the UCP2 expression. There is no relevant research involving the function of melatonin in LPS-induced cardiomyopathy, it is unclear whether the specificity expression of UCP2 in the different organs and the different models. This study is the first one to show the possible mechanism that melatonin may influence UCP2 expression in LPS-induced cardiomyopathy. It has been proven that melatonin protects mitochondria by scavenging reactive oxygen species (ROS), inhibiting the mitochondrial permeability transition pore (MPTP), and activating uncoupling proteins [38,41]. Also in our study, melatonin raised the level of UCP2 and prevented cardiomyocytes from LPS damage. 

The intrinsic of UCP2 is H^+^ channel locating on the inner mitochondrial membrane promoting the proton leak, which can influence △Ψm [42]. The meaning of uncoupling is the collapse of the △Ψm caused by proton leakage from the intermembrane space to the matrix [43]. It has been currently known that UCP2 overexpression can inhibit the depolarization of △Ψm which can decrease Ca^2+^ uptaking into the mitochondrial matrix [25,44,45]. Otherwise, the Ca^2+^ overload can result in ROS production and mitochondrial injury. Redundant ROS can stimulate the proton leak, so that leading the activity of UCP2 decreased and reducing the generation of ROS. UCP2 plays a protective role in the heart via this negative feedback loop [46]. Large experiments had represented that knockout of UCP2 results in a concomitant increase in mitochondrial ROS emission [47,48]. Our animal study found that mitochondrial membrane potential of the heart tissue of the WT + Melatonin + LPS group had higher than WT control group, WT + melatonin group and WT + LPS group. While, the fluorescence intensity of WT + LPS group and WT + Melatonin + LPS had significantly decreased when UCP2 knocked out. Furthermore, the level of oxidative stress increased significantly in UCP2 knockout group, while the level of oxidative stress decreased after melatonin intervention. Our findings from in vitro also explored △Ψm was totally collapsed, Ca^2+^ overload and excess ROS in genipin group, but had been recovered in melatonin group. Opening the mPTP is the result of the loss of △Ψm and Ca^2+^ overload, which will cause apoptosis protein releasing outside the mitochondrial membrane and induce the cell death [49,50]. Hence, UCP2 can inhibit the mPTP opening mediated by prevention of Ca^2+^ overload and ROS production, attenuating the cell apoptosis. Except for the ROS, ATP is the other indicator evaluating the mitochondrial function. Mitochondria is an ATP factory that can maintain cardiomyocytes metabolism and function. It is well known that ATP synthesis is depending on the coupling of oxidative phosphorylation. While, the coupling procedure relies on the proton leakage and is regulated by UCP2 [51]. Up to now, the relationship between UCP2 and ATP is still controversial. Some studies suggested that UCP2 can decrease the ATP generation [52,53,54], while others supported more UCP2 expression can lead elevated levels of ATP [55,56,57]. In this study, what can be identified was that LPS damaged the cells and reduced the production of ATP, melatonin can increase the UCP2 expression and recover ATP generation. 

Mild mitochondrial uncoupling has been proposed as a mechanism to protect cardiomyocytes from inflammatory injury via decreased ROS generation. Nevertheless, the concrete mechanism behind this effect is based on “uncoupling-to-survive” hypothesis [58,59]. The classical view is that the opening of mPTP and excess ROS generation can trigger programmed cell death/apoptosis. The difficulty of current research lies in reducing cell apoptosis or weakening the effect of necrotic cells on other healthy cells. Autophagy, like apoptosis, is another well-recognized cellular processes relying on lysosome. It is a process of degrading the contents of the encapsulated cells, thereby circulating the metabolic pathways and renewing some organelles [60]. Though protective effect of autophagy is still a debate, large amounts of research have proved its influence on cells and organs recovery [61,62]. One of the functions is as critical for mitochondrial homeostasis. Moreover, there are a variety of mechanisms by which the autophagy and apoptotic pathways can become intertwined to affect cell fate [60,63,64]. Up to now, several researches involving UCP2 regulation related to crosstalk with autophagy and apoptosis in nerve cell [65], cumulus cell [66], tubular epithelial cell [67], and hepatocyte [68]. However, to date, no study has been performed to confirm whether UCP2 overexpression can evoke autophagy and inhibit apoptosis in human cardiomyocytes. The results obtained in our study showed that apoptosis protein cytochrome C and caspase-3 increased in the UCP2 knocked out animal model and AC16 genipin group, but decreased under melatonin intervention. Apoptosis cells were significantly higher in UCP2 blocking group, suggesting that UCP2 plays a protective role against apoptosis. As for the further mechanism, we found the key protein Beclin-1 and LC-3β in autophagy “Beclin pathway” were obviously rise in UCP2 overexpression groups. According to the discovery in recent years, autophagy protein Beclin-1 is essential to regulate the switch between autophagy and apoptosis [69,70]. LC-3β acting as the downstream protein controls the autophagic flux and prevents the cells from “autophagic burst” [71]. Researchers have identified caspase-3 a predominant effector caspase in apoptosis can cleave Beclin-1 and inhibit autophagy activity [72,73]. For clarity, Beclin-1 and caspase-3 show the signaling connections between autophagy and apoptosis [74]. Until now, we speculate that UCP2 is crucial for protecting against sepsis-induced cardiomyopathy by promoting cardiomyocytes survival through autophagy induction and apoptosis reduction. And this is the first study presenting the position of UCP2 in crosstalk between autophagy and apoptosis. 

## 5. Conclusions

In summary, our study demonstrates that UCP2 may play a protecting role in cardiomyocytes against LPS. The reason for this is that UCP2 could regulate the balance between autophagy and apoptosis, which contribute to maintain cardiomyocytes activity. Additionally, melatonin maybe a potential regulator for UCP2 balance the autophagy and apoptosis.

## References

[1] Singer M., Deutschman C.S., Seymour C.W., Shankar-Hari M., Annane D., Bauer M., Bellomo R., Bernard G.R., Chiche J.D., Coopersmith C.M. (2016). The third international consensus definitions for sepsis and septic shock (sepsis-3). JAMA.

[2] Rudiger A., Singer M. (2013). The heart in sepsis: From basic mechanisms to clinical management. Curr. Vasc. Pharmacol..

[3] Flynn A., Chokkalingam Mani B., Mather P.J. (2010). Sepsis-induced cardiomyopathy: A review of pathophysiologic mechanisms. Heart Fail. Rev..

[4] Rudiger A., Singer M. (2007). Mechanisms of sepsis-induced cardiac dysfunction. Crit. Care Med..

[5] Flierl M.A., Rittirsch D., Huber-Lang M.S., Sarma J.V., Ward P.A. (2008). Molecular events in the cardiomyopathy of sepsis. Mol. Med..

[6] Liu Y.C., Yu M.M., Shou S.T., Chai Y.F. (2017). Sepsis-induced cardiomyopathy: Mechanisms and treatments. Front. Immunol..

[7] Yu X.X., Barger J.L., Boyer B.B., Brand M.D., Pan G., Adams S.H. (2000). Impact of endotoxin on ucp homolog mrna abundance, thermoregulation, and mitochondrial proton leak kinetics. Am. J. Physiol. Endocrinol. Metab..

[8] Ricquier D., Miroux B., Cassard-Doulcier A.M., Levi-Meyrueis C., Gelly C., Raimbault S., Bouillaud F. (1999). Contribution to the identification and analysis of the mitochondrial uncoupling proteins. J. Bioenerg. Biomembr..

[9] Harper M.E., Gerrits M.F. (2004). Mitochondrial uncoupling proteins as potential targets for pharmacological agents. Curr. Opin. Pharmacol..

[10] Chen Z.J., Song Y.B., Wang H.L., Wang Y., Lv J.J., Che D., Zeng Q.Y. (2014). Effect of ucp2-sirna on inflammatory response of cardiomyocytes induced by septic serum. Zhongguo Dang Dai Er Ke Za Zhi.

[11] Hoshovs’ka Iu V., Shymans’ka T.V., Sahach V.F. (2009). Effect of UCP2 activity inhibitor genipin on heart function of aging rats. Fiziol. Zhurnal.

[12] Safari F., Bayat G., Shekarforoush S., Hekmatimoghaddam S., Anvari Z., Moghadam M.F., Hajizadeh S. (2014). Expressional profile of cardiac uncoupling protein-2 following myocardial ischemia reperfusion in losartan- and ramiprilat-treated rats. J. Renin Angiotensin Aldost. Syst..

[13] Xu J., Nie H.G., Zhang X.D., Tian Y., Yu B. (2011). Down-regulated energy metabolism genes associated with mitochondria oxidative phosphorylation and fatty acid metabolism in viral cardiomyopathy mouse heart. Mol. Biol. Rep..

[14] Acuna-Castroviejo D., Rahim I., Acuna-Fernandez C., Fernandez-Ortiz M., Solera-Marin J., Sayed R.K.A., Diaz-Casado M.E., Rusanova I., Lopez L.C., Escames G. (2017). Melatonin, clock genes and mitochondria in sepsis. Cell. Mol. Life Sci..

[15] Galley H.F., Lowes D.A., Allen L., Cameron G., Aucott L.S., Webster N.R. (2014). Melatonin as a potential therapy for sepsis: A phase i dose escalation study and an ex vivo whole blood model under conditions of sepsis. J. Pineal Res..

[16] Jimenez-Aranda A., Fernandez-Vazquez G., Campos D., Tassi M., Velasco-Perez L., Tan D.X., Reiter R.J., Agil A. (2013). Melatonin induces browning of inguinal white adipose tissue in zucker diabetic fatty rats. J. Pineal Res..

[17] Ho J., Yu J., Wong S.H., Zhang L., Liu X., Wong W.T., Leung C.C., Choi G., Wang M.H., Gin T. (2016). Autophagy in sepsis: Degradation into exhaustion?. Autophagy.

[18] Li M., Gao P., Zhang J. (2016). Crosstalk between autophagy and apoptosis: Potential and emerging therapeutic targets for cardiac diseases. Int. J. Mol. Sci..

[19] Bravo-San Pedro J.M., Kroemer G., Galluzzi L. (2017). Autophagy and mitophagy in cardiovascular disease. Circ. Res..

[20] Goldenthal M.J. (2016). Mitochondrial involvement in myocyte death and heart failure. Heart Fail. Rev..

[21] Turdi S., Han X., Huff A.F., Roe N.D., Hu N., Gao F., Ren J. (2012). Cardiac-specific overexpression of catalase attenuates lipopolysaccharide-induced myocardial contractile dysfunction: Role of autophagy. Free Radic. Biol. Med..

[22] Song J., Kang S.M., Lee K.M., Lee J.E. (2015). The protective effect of melatonin on neural stem cell against lps-induced inflammation. Biomed. Res. Int..

[23] Kiers D., van der Heijden W.A., van Ede L., Gerretsen J., de Mast Q., van der Ven A.J., El Messaoudi S., Rongen G.A., Gomes M., Kox M. (2017). A randomised trial on the effect of anti-platelet therapy on the systemic inflammatory response in human endotoxaemia. Thromb. Haemost..

[24] Lee I.C., Kim D.Y., Bae J.S. (2017). Sulforaphane reduces hmgb1-mediated septic responses and improves survival rate in septic mice. Am. J. Chin. Med..

[25] Zheng G., Lyu J., Liu S., Huang J., Liu C., Xiang D., Xie M., Zeng Q. (2015). Silencing of uncoupling protein 2 by small interfering rna aggravates mitochondrial dysfunction in cardiomyocytes under septic conditions. Int. J. Mol. Med..

[26] Joshi M.S., Julian M.W., Huff J.E., Bauer J.A., Xia Y., Crouser E.D. (2006). Calcineurin regulates myocardial function during acute endotoxemia. Am. J. Respir. Crit. Care Med..

[27] Lopez L.C., Escames G., Tapias V., Utrilla P., Leon J., Acuna-Castroviejo D. (2006). Identification of an inducible nitric oxide synthase in diaphragm mitochondria from septic mice: Its relation with mitochondrial dysfunction and prevention by melatonin. Int. J. Biochem. Cell Biol..

[28] Escames G., Lopez L.C., Ortiz F., Lopez A., Garcia J.A., Ros E., Acuna-Castroviejo D. (2007). Attenuation of cardiac mitochondrial dysfunction by melatonin in septic mice. FEBS J..

[29] Escames G., Lopez L.C., Tapias V., Utrilla P., Reiter R.J., Hitos A.B., Leon J., Rodriguez M.I., Acuna-Castroviejo D. (2006). Melatonin counteracts inducible mitochondrial nitric oxide synthase-dependent mitochondrial dysfunction in skeletal muscle of septic mice. J. Pineal Res..

[30] Zhang H., Liu D., Wang X., Chen X., Long Y., Chai W., Zhou X., Rui X., Zhang Q., Wang H. (2013). Melatonin improved rat cardiac mitochondria and survival rate in septic heart injury. J. Pineal Res..

[31] Oliveira M.T., Garesse R., Kaguni L.S. (2010). Animal models of mitochondrial DNA transactions in disease and ageing. Exp. Gerontol..

[32] Donadelli M., Dando I., Fiorini C., Palmieri M. (2014). UCP2, a mitochondrial protein regulated at multiple levels. Cell. Mol. Life Sci..

[33] Jiang Z.M., Yang Q.H., Zhu C.Q. (2017). UCP2 in early diagnosis and prognosis of sepsis. Eur. Rev. Med. Pharmacol. Sci..

[34] Wang X., Liu D., Chai W., Long Y., Su L., Yang R. (2015). The role of uncoupling protein 2 during myocardial dysfunction in a canine model of endotoxin shock. Shock.

[35] Roshon M.J., Kline J.A., Thornton L.R., Watts J.A. (2003). Cardiac UCP2 expression and myocardial oxidative metabolism during acute septic shock in the rat. Shock.

[36] Shang Y., Liu Y., Du L., Wang Y., Cheng X., Xiao W., Wang X., Jin H., Yang X., Liu S. (2009). Targeted expression of uncoupling protein 2 to mouse liver increases the susceptibility to lipopolysaccharide/galactosamine-induced acute liver injury. Hepatology.

[37] Wang Q., Wang J., Hu M., Yang Y., Guo L., Xu J., Lei C., Jiao Y., Xu J. (2016). Uncoupling protein 2 increases susceptibility to lipopolysaccharide-induced acute lung injury in mice. Mediat. Inflamm..

[38] Tan D.X., Manchester L.C., Qin L., Reiter R.J. (2016). Melatonin: A mitochondrial targeting molecule involving mitochondrial protection and dynamics. Int. J. Mol. Sci..

[39] Navarro-Alarcon M., Ruiz-Ojeda F.J., Blanca-Herrera R.M., MM A.S., Acuna-Castroviejo D., Fernandez-Vazquez G., Agil A. (2014). Melatonin and metabolic regulation: A review. Food Funct..

[40] Agil A., El-Hammadi M., Jimenez-Aranda A., Tassi M., Abdo W., Fernandez-Vazquez G., Reiter R.J. (2015). Melatonin reduces hepatic mitochondrial dysfunction in diabetic obese rats. J. Pineal Res..

[41] Fernandez Vazquez G., Reiter R.J., Agil A. (2018). Melatonin increases brown adipose tissue mass and function in zucker diabetic fatty rats: Implications for obesity control. J. Pineal Res..

[42] Pecqueur C., Alves-Guerra M.C., Gelly C., Levi-Meyrueis C., Couplan E., Collins S., Ricquier D., Bouillaud F., Miroux B. (2001). Uncoupling protein 2, in vivo distribution, induction upon oxidative stress, and evidence for translational regulation. J. Biol. Chem..

[43] Moon J.S., Lee S., Park M.A., Siempos I.I., Haslip M., Lee P.J., Yun M., Kim C.K., Howrylak J., Ryter S.W. (2015). UCP2-induced fatty acid synthase promotes NLRP3 inflammasome activation during sepsis. J. Clin. Investig..

[44] Teshima Y., Akao M., Jones S.P., Marban E. (2003). Uncoupling protein-2 overexpression inhibits mitochondrial death pathway in cardiomyocytes. Circ. Res..

[45] Li N., Wang J., Gao F., Tian Y., Song R., Zhu S.J. (2010). The role of uncoupling protein 2 in the apoptosis induced by free fatty acid in rat cardiomyocytes. J. Cardiovasc. Pharmacol..

[46] Akhmedov A.T., Rybin V., Marin-Garcia J. (2015). Mitochondrial oxidative metabolism and uncoupling proteins in the failing heart. Heart Fail. Rev..

[47] Lee S.C., Robson-Doucette C.A., Wheeler M.B. (2009). Uncoupling protein 2 regulates reactive oxygen species formation in islets and influences susceptibility to diabetogenic action of streptozotocin. J. Endocrinol..

[48] Arsenijevic D., Onuma H., Pecqueur C., Raimbault S., Manning B.S., Miroux B., Couplan E., Alves-Guerra M.C., Goubern M., Surwit R. (2000). Disruption of the uncoupling protein-2 gene in mice reveals a role in immunity and reactive oxygen species production. Nat. Genet..

[49] Liu D., Yi B., Liao Z., Tang L., Yin D., Zeng S., Yao J., He M. (2014). 14–3-3gamma protein attenuates lipopolysaccharide-induced cardiomyocytes injury through the bcl-2 family/mitochondria pathway. Int. Immunopharmacol..

[50] Crompton M., Costi A., Hayat L. (1987). Evidence for the presence of a reversible Ca^2+^-dependent pore activated by oxidative stress in heart mitochondria. Biochem. J..

[51] Le Minh K., Kuhla A., Abshagen K., Minor T., Stegemann J., Ibrahim S., Eipel C., Vollmar B. (2009). Uncoupling protein-2 deficiency provides protection in a murine model of endotoxemic acute liver failure. Crit. Care Med..

[52] Fang W.J., Wang C.J., He Y., Zhou Y.L., Peng X.D., Liu S.K. (2017). Resveratrol alleviates diabetic cardiomyopathy in rats by improving mitochondrial function through PGC-1alpha deacetylation. Acta Pharmacol. Sin..

[53] Diao J.Y., Wei J., Yan R., Lin L., Li H. (2016). Effect of uncoupling protein 2 on high-glucose induced mitochondrial damage and apoptosis of cardiomyocytes. Zhonghua Yi Xue Za Zhi.

[54] Chen G.G., Yan J.B., Wang X.M., Zheng M.Z., Jiang J.P., Zhou X.M., Cai B., Shen Y.L. (2016). Mechanism of uncoupling protein 2mediated myocardial injury in hypothermic preserved rat hearts. Mol. Med. Rep..

[55] Su J., Liu J., Yan X.Y., Zhang Y., Zhang J.J., Zhang L.C., Sun L.K. (2017). Cytoprotective effect of the UCP2-sirt3 signaling pathway by decreasing mitochondrial oxidative stress on cerebral ischemia-reperfusion injury. Int. J. Mol. Sci..

[56] Kretzschmar C., Roolf C., Timmer K., Sekora A., Knubel G., Escobar H.M., Jaster R., Muller S., Fuellen G., Kohling R. (2016). Uncoupling protein 2 deficiency results in higher neutrophil counts and lower β-cell counts during aging in mice. Exp. Hematol..

[57] Cheng G., Polito C.C., Haines J.K., Shafizadeh S.F., Fiorini R.N., Zhou X., Schmidt M.G., Chavin K.D. (2003). Decrease of intracellular atp content downregulated UCP2 expression in mouse hepatocytes. Biochem. Biophys. Res. Commun..

[58] Brand M.D. (2000). Uncoupling to survive? The role of mitochondrial inefficiency in ageing. Exp. Gerontol..

[59] Rose G., Crocco P., De Rango F., Montesanto A., Passarino G. (2011). Further support to the uncoupling-to-survive theory: The genetic variation of human UCP genes is associated with longevity. PLoS ONE.

[60] Song S., Tan J., Miao Y., Li M., Zhang Q. (2017). Crosstalk of autophagy and apoptosis: Involvement of the dual role of autophagy under er stress. J. Cell. Physiol..

[61] Wang R., Shen Z., Yang L., Yin M., Zheng W., Wu B., Liu T., Song H. (2017). Protective effects of heme oxygenase-1-transduced bone marrow-derived mesenchymal stem cells on reducedsize liver transplantation: Role of autophagy regulated by the erk/mtor signaling pathway. Int. J. Mol. Med..

[62] Gunst J., Derese I., Aertgeerts A., Ververs E.J., Wauters A., Van den Berghe G., Vanhorebeek I. (2013). Insufficient autophagy contributes to mitochondrial dysfunction, organ failure, and adverse outcome in an animal model of critical illness. Crit. Care Med..

[63] Liu G., Pei F., Yang F., Li L., Amin A.D., Liu S., Buchan J.R., Cho W.C. (2017). Role of autophagy and apoptosis in non-small-cell lung cancer. Int. J. Mol. Sci..

[64] Kemp M.G. (2017). Crosstalk between apoptosis and autophagy: Environmental genotoxins, infection, and innate immunity. J. Cell Death.

[65] Marques-Aleixo I., Santos-Alves E., Balca M.M., Rizo-Roca D., Moreira P.I., Oliveira P.J., Magalhaes J., Ascensao A. (2015). Physical exercise improves brain cortex and cerebellum mitochondrial bioenergetics and alters apoptotic, dynamic and auto(mito)phagy markers. Neuroscience.

[66] Ge H., Zhang F., Duan P., Zhu N., Zhang J., Ye F., Shan D., Chen H., Lu X., Zhu C. (2017). Mitochondrial uncoupling protein 2 in human cumulus cells is associated with regulating autophagy and apoptosis, maintaining gap junction integrity and progesterone synthesis. Mol. Cell. Endocrinol..

[67] Zhou Y., Cai T., Xu J., Jiang L., Wu J., Sun Q., Zen K., Yang J. (2017). UCP2 attenuates apoptosis of tubular epithelial cells in renal ischemia-reperfusion injury. Am. J. Physiol. Renal Physiol..

[68] Lou J., Wang Y., Wang X., Jiang Y. (2014). Uncoupling protein 2 regulates palmitic acid-induced hepatoma cell autophagy. Biomed. Res. Int..

[69] Salminen A., Kaarniranta K., Kauppinen A. (2013). Beclin 1 interactome controls the crosstalk between apoptosis, autophagy and inflammasome activation: Impact on the aging process. Ageing Res. Rev..

[70] Marquez R.T., Xu L. (2012). Bcl-2:Beclin 1 complex: Multiple, mechanisms regulating autophagy/apoptosis toggle switch. Am. J. Cancer Res..

[71] Scherz-Shouval R., Weidberg H., Gonen C., Wilder S., Elazar Z., Oren M. (2010). P53-dependent regulation of autophagy protein LC3 supports cancer cell survival under prolonged starvation. Proc. Natl. Acad. Sci. USA.

[72] Mazumder S., Plesca D., Almasan A. (2008). Caspase-3 activation is a critical determinant of genotoxic stress-induced apoptosis. Methods Mol. Biol..

[73] Zhu Y., Zhao L., Liu L., Gao P., Tian W., Wang X., Jin H., Xu H., Chen Q. (2010). Beclin 1 cleavage by caspase-3 inactivates autophagy and promotes apoptosis. Protein Cell.

[74] Ye L.X., Yu J., Liang Y.X., Zeng J.S., Huang R.X., Liao S.J. (2014). Beclin 1 knockdown retards re-endothelialization and exacerbates neointimal formation via a crosstalk between autophagy and apoptosis. Atherosclerosis.

